# Prevalence and associated factors of *Tunga penetrans* infestation among 5-14-year-olds in rural Ethiopia

**DOI:** 10.1371/journal.pone.0259411

**Published:** 2021-10-29

**Authors:** Aiggan Tamene

**Affiliations:** Environmental Health Unit, School of Public Health, College of Medicine and Health Sciences, Wachemo University, Hossaena, Ethiopia; University of Cape Town Faculty of Health Sciences, SOUTH AFRICA

## Abstract

**Background:**

Tunga penetrans, also known as sand flea, causes Tungiasis in humans and animals. Despite its notoriety as an entomological problem, however, the ectoparasite receives little consideration from public health professionals. It is against this background that this article aims to assess the prevalence of and factors associated with *Tunga penetrans* infestation among 5-14-year-olds in rural Western Ethiopia.

**Methods:**

From November to December 2020, 487 children aged 5 to 14 were selected from four primary schools in a cross-sectional study using systematic random sampling. Clinical exams, Interviews with parents/guardians and observations of the housing and classroom environments were used to collect data. Descriptive statistics and multivariable regression were used to characterize the data and identify factors associated with *Tunga penetrans* infestation.

**Result:**

*Tunga penetrans* infestation (Tungiasis) was diagnosed in 138 of the 487 children examined, placing the prevalence at 28.3% (95% CI: 24.2%, 32.2%). Mud plastered walls [AOR: 5.83, % CI (3.44–9.88)], having cats in the house [AOR: 5.91, 95% CI (3.51–10.11)], not having separated sleeping quarters for animals [AOR: 4.60, 95% CI (2.69–7.86)], using self-supplied water [AOR: 6.30, 95% CI (3.33–11.93)], walking>30 minutes to school [AOR: 2.37, 95% CI (1.48–3.80)] were associated with Tungiasis.

**Conclusion:**

In one way or another, several of the identified factors were linked to poverty. Improved house wall materials, fumigation of mud-plastered houses, dusting or spraying insecticides on domestic animals (such as cats), improved access to water, community education about keeping animals separated from living spaces, and hygiene promotion are all needed, with a focus on locally available, low-cost technologies that the poorest families can afford.

## Introduction

Tungiasis is a zoonosis caused by female *Tunga penetrans* and *Tunga trimamillata* sand fleas embedded in the skin [1]. It is a Neglected Tropical Disease that is prevalent in resource-poor rural areas throughout Sub-Saharan Africa, the Caribbean, and South America [2]. Children aged 5 to 14 and the elderly are the most affected, with prevalence rates as high as 53% [3].

Once lodged in the skin, the flea matures, most commonly on the toes, soles, and heels, however, infestations can also occur in the hands, elbows, genital and anal regions. It takes up to five weeks for the flea to grow to the size of a pea, then it produces and releases eggs before dying [2]. Hence, morbidity is related to an intense inflammatory response triggered by the development of sand fleas embedded in the epidermis [1].

Although Tungiasisis a self-limited infestation, complications are common in endemic areas [2]. Many patients report severe pain and inflammation, and fissures commonly hinder individuals from walking normally [4]. Sequels include deformation and loss of toenails, as well as the deformation of digits. The sore in the skin caused by the protruding rear end of the flea is an entry point for pathogenic microorganisms [5]. Super infected lesions lead to the formation of pustules, and suppuration. It can also lead to tetanus in people who haven’t been vaccinated [6].

Poor personal hygiene, poor housing and residential environment cleanliness, and a lack of footwear have all been associated to Tungiasis infection [6–8]. Infestation is also linked to living with reservoir domestic animals [such as cats, dogs, and pigs] [2]. Tungiasis in animals is significantly linked to human Tungiasis, with the presence of the disease in animals increasing the likelihood of infection in humans by a factor of six [6]. Once infested with *Tunga penetrans*, children find it difficult to travel to school and attend classes due to the persistent symptoms and social stigma connected with the infestation [7].

Health practitioners in the developing world have grown more aware of the burden of neglected tropical illnesses in recent years [9]. Despite its reputation as an entomological nuisance, however, the ectoparasite receives little attention from public health officials. The Limu Saka district is one of the largest districts in Western Ethiopia. Anecdotal evidence suggests that *Tunga penetrans* infestation is still a major nuisance for the local population. Hitherto, no systematic investigations have been conducted on the prevalence and associated factors of Tungiasis in rural agro-pastoralist communities of Western Ethiopia.

## Materials and methods

### Study area and setting

A cross-sectional study was carried out in the Oromia Region, one of Ethiopia’s ten federal states, from November to December 2020. Limu Saka district is situated 450 kilometres west of Addis Ababa, Ethiopia’s capital, and is divided into 12 rural and 4 urban Kebeles (the lowest administrative unit in Ethiopia). This district has a total population of 189,463, with 95,869 men and 93,594 women; urban dwellers account for 5,185 people or 2.74% of the total population [10]. Farmers and livestock breeders make up the majority of the district’s residents. Among the animals raised are cows, pigs, hens, and domestic pets.

### Sample size and sampling procedure

The sample size required to estimate the prevalence and correlates of *Tunga penetrans* infestation in the Limu Saka district was calculated using the Epi Info 7 software. The sample sizes for objectives one and two were determined separately based on different assumptions, using a formula for a single population for the first objective and a formula for a double population for the second. Finally, for both objectives, the sample size (n = 492) with assumptions of an estimated prevalence of 25% [11], a 95% level of confidence, a 5% margin of error, a design effect of 1.5, and an expected non-response rate of 10% was found to be the largest.

To achieve the study’s objective, a multi-stage sampling technique was employed. In the first phase, 4 (30%) of the total Kebeles were chosen at random from the district’s 12 rural Kebele administrations. In the selected Kebeles, there were a total of seven schools. The goal of this study was to undertake a Tungiasis prevalence investigation in very rural and dispersed settlements as Tungiasis investigations had previously been conducted in urban, semi-urban, and compact rural settlements.

Accordingly, relevant school districts in very remote and dispersed sections of the district were chosen after consultations with district officials. The four most rural schools were chosen to offer crucial information that could not be collected through other approaches (Urban slums, suburban, and compact rural settlements). The goal of this study was to undertake a Tungiasis prevalence investigation in very rural and dispersed settlements as Tungiasis investigations had previously been conducted in urban, semi-urban, and compact rural settlements. Accordingly, 30% of the schools in very remote and dispersed sections of the district were randomly chosen. In the third step, the total sample size, i.e.,492was allocated to the selected primary schools proportional to the schools’ pupil numbers ((Sample size/Population size) x Stratum size) (Table 1).

**Table 1:** The distribution of children attending selected primary schools Limu Saka, Ethiopia, 2021.

Primary schools	Total number of pupils	Selected	Proportional allocation	Skip interval (K-value)
Primary school A	1303	170	0.13*1303	8
Primary school B	847	111	0.13*847	8
Primary school C	1002	132	0.13*1002	8
Primary school D	603	79	0.13*603	8
Total	3755	492		

Finally, systematic random sampling with a skip interval approach was used to draw study participants from each selected school. The lists of students present in each class room on the day of visit were taken from the record offices of their respective schools. They were then allocated ordinal numbers based on their alphabetical order. The skip interval (K) was calculated by dividing the total number of students in each chosen school by the sample sizes proportionally allocated. Using a random starting point, every eighth student on the sampling frame was chosen for data collection until the completion.

The selection of study participants emphasized unrelated subsets of individuals where only one member of a family is included. If more than one sibling was chosen, one sibling per sibship was chosen at random. If the guardians of the chosen child declined to participate in the study, the next child on the sample frame was chosen.

### Data collection

The district initiated the Rural Health Extension Program 16 years ago, with a focus on improving environmental and community health [12]. Since then, two health extension personnel have been assigned to provide door-to-door education in each Kebele. After receiving training on data collection tools and procedures, these health extension workers collected data in the present study. Physical assessment of the children, interviews with their parents/guardians, and observation of the children’s home and school environment were used to collect data.

#### Physical examination

*Tunga Penetrans* infestation (Tungiasis) was evaluated in all students for whom informed consent was provided. The students’ feet were thoroughly washed with soap in a bucket before the clinical assessment. Each person was then screened for Tungiasis using a standardized procedure [13]. This assessment took place at their residences.

#### Structured interviews

Data were obtained via face-to-face interviews with participants using a pretested and closed-ended questionnaire developed by the investigator (S1 File). Primary care providers were asked about their educational backgrounds, household conditions, water sources and water access, hygiene practices, livestock, and domestic animals. In addition, observations were made about the physical layout of the classrooms where the children learnt, students’ hygiene, and the type of shoes they wore if any.

### Study variables

The infestation with *Tunga penetrans* was used as the dependent variable in this study. Socio-demographic characteristics (sex of the child, age of the child, time spent commuting to school, maternal education, income, family size), household conditions (household water supply, household latrine system, household waste disposal system, nature of household roof material, nature of household floor material), environmental conditions of school (floor type, wall type), and behavioural characteristics (frequency of washing feet, frequency of washing feet with soap, the habit of wearing shoes) were all independent variables.

### Operational definition

#### *Tunga penetrans* infestation (Tungiasis)

Based on the Fortaleza classification, the results were deemed pathognomonic for *T*.*penetrans* infestation at the time of the examination if nodules with black centers, suppurative ulcers or punctiform cavities, itching spots, trouble walking, oedema, and skin redness around lesions, loss of toenails or deformed nails were detected [11].

#### Manipulated lesions

Lesions that have been manipulated with a sharp instrument (by the patient or their caregiver) to remove the parasite that has become embedded [11].

#### The intensity of lesions

The severity of the lesions can be categorized as light (1–5 lesions), moderate (6–30 lesions), or severe (>30 lesions) [14].

#### Absolute poverty

Absolute poverty is characterized as a family’s inability to meet the food budget, as well as a separate allowance for non-food products, per the poor’s spending patterns. For 2020, the absolute poverty line is set at 163.09 birr per week per adult equivalent (1 United States Dollar equals 40.25 Ethiopian birr) [15].

#### Improved latrine facility

Pit latrine with a slab, ventilated improved pit latrine, composting toilet, flush, pour/flush facility connected to a piped sewer system/septic tank/pit [16].

#### Non-improved latrine facility

Flush or pour/flush facility not connected to a piped sewer system/septic tank/pit, pit latrine without a slab/ open pit, hanging latrine/bucket latrine [16].

#### Self-supplied (self-collected) water consumer

A person/household that withdraws water from a groundwater or surface-water source rather than using a piped supply. Water from domestic wells, rainwater, rivers, and so on… is deemed to be self-supplied water [17].

### Data quality assurance

The investigator employed four data collectors and one field supervisor. A professional translator produced forward and reverse translations of the questionnaire. The supervisor and data collectors received two days of rigorous training on data collection instruments and techniques. The training included both a theoretical and a practical session, during which the data collectors visited another school in a neighbouring district and practiced conducting some of the physical inspection tasks. The questionnaire was then pre-tested in another school in the neighbouring district on 5% of the overall sample size. The tool was finalized after the pre-test and necessary changes. The results of the pre-test were excluded from the main analysis.

### Data management and analysis

Before being entered into the computer, all the data collected via questionnaires were reviewed for errors and coded. The data was inserted into Epi info version 7 software using a data entry template; the data was then cleaned and analyzed using the statistical package for social sciences (SPSS V.20). Descriptive results were presented as frequency tables, percentages, and proportions with a 95% confidence level (CI). Factors associated with Tungiasis were identified using binary logistic regression. The logistic regression analysis commenced with a crude analysis in which each potential determinant was independently investigated for a correlation to the outcome variable. In the initial multivariable model, variables with p-values up to 0.25 in the crude analysis and those considered significant in previous studies were included. This cut-off point was chosen to minimize a superfluous number of variables and an unstable estimate in the multivariable regression.

In the multivariable analysis, variables with a p-value of less than 0.05 were considered statistically significant and presented by an Adjusted Odds Ratio (AOR) with a 95%% confidence interval. In the course of the analysis, multicollinearity among the explanatory variables was tested using the variance inflation factor (VIF<10), suggesting that one independent variable was not explained by another in the model. The Hosmer-Lemeshow goodness of fit test (p-value = 0.43) was used to determine model fitness. At a p-value of 0.05, the Hosmer-Lemeshow test should be insignificant; signalling that the variable entered fits the model.

This study was authorized and carried out per the Helsinki Declaration’s principles. The Institutional Review Board of Wachemo University College of Medicine and Health Sciences granted ethical approval. An approval letter from the Oromia Regional Health Bureau and the Limu Saka District Health Bureau was obtained before data collection. The parents/guardians of the children gave their written informed consent. Participants’ privacy and confidentiality were ensured before, during, and after data collection. Soap, sterile needle, surgical blade, and Vaseline ointment were given to all children who took part in the study.

## Results

### Study demographics

487 parents and caregivers completed the interviews, giving the study a response rate of 98.9%. The children’s average age was 10.7 years old, with a standard deviation of 1.8 years. In terms of gender, 224 students (46.0%) were male, while 263 students (54.1%) were female. When going to or returning from school, the majority of the students, i.e., 295 (60.6%), walked 30 minutes or less. 450 (92.4%) of the children lived in households with five or fewer individuals, while four (0.8%) lived in households with more than ten people. Furthermore, 199 (40.9%) of the primary caregivers had no formal schooling. Concerning monthly income, 212 (43.5%) of the households were in poverty (Table 2).

**Table 2:** Socio-demographic characteristics of study participants, Limu Saka, Ethiopia, 2021.

Variables	Categories	Frequency (n = 487) (%)
Sex	Male	224 (46.0%)
	Female	263 (54.0%)
Age	5–9 years	150 (30.8%)
	10–14 years	337 (69.2%)
Walk time to school	<30 minutes each way	295 (60.6%)
	>30 minutes each way	192 (39.4%)
Maternal Education	No formal education	199 (40.9%)
	Primary schooling (grades 1–8)	166 (32.0%)
	Secondary schooling (grades 9–10)	70 (14.4%)
	Vocational training and above*	62 (12.7%)
Family size	≤ 5	450 (92.4%)
	6–10	33 (6.8%)
	>10	4 (0.8%)
Family Income	≤101.28 birr	52 (10.7%)
	101.29–163.09 birr	212 (43.5%)
	>163.09 birr	223 (45.8%)

* Vocational training (grade 10+2), diploma, degree, masters, etc…

### Household characteristics of participants

Smeared mud was used as a wall plastering material in 433 (88.9%) of the participants’ homes, while 238 (48.9%) used it on their floors. A corrugated sheet was used as a roofing material by the majority of the respondents, i.e., 335 (68.8%). In terms of household waste disposal, 415 (85.2%) of the households dumped their trash in the open. Non-improved latrines accounted for 142 (29.2%) total latrines. Traditional pit latrines without a slab and flush latrines not connected to a sewer and septic tank accounted for 80 (56.3%) and 54 (38.0%) of the unimproved latrines, respectively.

While 400 people (82.1%) had piped water installed in their home, 423 people (86.9%) had to travel more than 30 minutes round trip to get water citing concerns with water supply reliability and water point functionality. The majority of respondents had goats (85.4%), cows61 (86.9%) and chickens (73.1%) present in the compound at the time of the survey but only 62 (16.8%) reported sharing sleeping quarters with their animals (Table 3).

**Table 3:** Household characteristics of study participants, Limu Saka, Ethiopia, 2021.

Variables	Category	Frequency (n = 487) (%)
Materials of House wall	Stone/ Cement	54 (11.1%)
	Smeared mud	433 (88.9%)
Materials of House floor	Mud	238 (48.9%)
	Sand	12 (2.5%)
	Stone/cement	237 (48.7%)
Materials of House roof	Thatched	152 (31.2%)
	Corrugated sheet	335 (68.8%)
Household waste disposal	Open dumping	415 (85.2%)
	Burn/burry	57 (11.7%)
	Use a collection service	15 (3.1%)
Household latrine facility	Open defecation	103 (21.2%)
	Non-improved latrine	142 (29.2%)
	Improved, shared latrine	139 (28.5%)
	Improved, not shared latrine	103 (21.2%)
Water source	Piped into dwelling	400 (82.1%)
	Community tap/ Community wells	20 (4.15%)
	Self-supplied	67 (13.75%)
Time travelled to fetch water	≤30 minutes	64 (13.1%)
	>30 minutes	423 (86.9%)
The sleeping situation of the child		
	Floor	137 (28.1%)
	Raised bed	350 (71.9%)
Domestic animals in the compound		
Dog		
	Yes	238 (48.9%)
	No	249 (51.1%)
Cats		
	Yes	110 (22.6%)
	No	377 (77.4%)
Goats		
	Yes	416 (85.4%)
	No	71 (14.6%)
Cows		
	Yes	423 (86.9%)
	No	64 (13.1%)
Chicken		
	Yes	356 (73.1%)
	No	131 (26.9%)
Share sleeping quarters with animals		
	Yes	82 (16.8%)
	No	405 (83.2%)

### Foot hygiene and footwear habits of participants

In terms of their footwear patterns, 8 (1.6%) of the children walked barefoot all of the time, while 267 (54.8%) wore shoes daily. During the seven-day week, 241 (49.5%) of the children washed their feet less than once per day; those who used soap every time they washed their feet had a much lower percentage of 26.7%. When their feet were examined, more than half of the children (58.3%) had dusty or unwashed feet. The study participants were also inspected at their schools to determine the type of shoe they wore to school that day; 102 (20.9%) of the students wore closed shoes, 337 (69.2%) wore open shoes, and 48 (9.9%) were barefoot (Table 4).

**Table 4:** Foot hygiene and footwear habits of participants, Limu Saka, Ethiopia, 2021.

Variables	Category	Frequency (n = 487) (%)
Footwear habit	Never (always barefoot)	8 (1.6%)
	Sometimes	212 (43.5%)
	Always	267 (54.8%)
Feet washing	> 2 times a day	12 (2.5%)
	Once per day	234 (48.0%)
	Less than once per day	241 (49.5%)
Feet washing with soap	Never	30 (6.2%)
	Sometimes	327 (67.1%)
	Always	130 (26.7%)
Feet hygiene on inspection	Dirty feet	284 (58.3%)
	Clean feet	203 (41.7%)
Shoes worn to school	Closed shoes	102 (20.9%)
	Open shoes	337 (69.2%)
	No shoes	48 (9.9%)

### Observational findings in the primary schools

The classrooms from where the study participants were chosen were also inspected. 24 Classrooms were investigated for roofing, flooring, and wall plastering problems. The school-based observation aimed to supplement the household-based analysis and see whether there are any particular school factors linked to the disease, as well as if school-based treatment and prevention interventions are a viable choice. Students taught in rooms with smooth concrete floors made up 21.2% of all screened students, while students taught in rooms with cracked concrete, smeared mud, and natural soil accounted for 28.4%, 29.2%, and 21.2%, respectively. 6.8% of the students sampled were thought in classrooms with mud walls. Just 14% of the students studied in a classroom with a grass roof. Each class had a surface area of 36m^2^ and could accommodate an average of 55 students.

### Prevalence and distribution of *T*.*penetrans* infestation

*Tunga penetrans* infestation (Tungiasis) was diagnosed in 138 of the 487 children examined, placing the prevalence of Tungiasis among 5-14-year-olds in Limu Saka district at 28.3% (95% CI: 24.2%, 32.2%). Among those with Tungiasis males accounted for 53.6%. Of the 138 people who had an infestation, 118 (85.8%) had *Tunga penetrans* lesions only on their feet, 16 (11.6%) had lesions on their hands, and 4 (2.8%) had lesions on their hands and feet contemporaneously.

### Intensity of *T*.*penetrans* lesions

A total of 1837 lesions were discovered on 138 children, with a mean parasite level of 6.1 lesions per child. Out of all the infected people, 41 (29.7%) had a mild infestation (1–5 lesions), 69 (50%) had a moderate infestation (6–30 lesions), and 28 (20.3%) had a severe infestation (>30 lesions). Deformity or loss of toenails was the most prevalent complication of *T*.*penetrans* infection, accounting for 29 (37.6%), while desquamation, hyperkeratosis, and fissures accounted for 11 (14.2%), 21 (27.5%), and 16 (20.7%), respectively.

**Fig 1:**
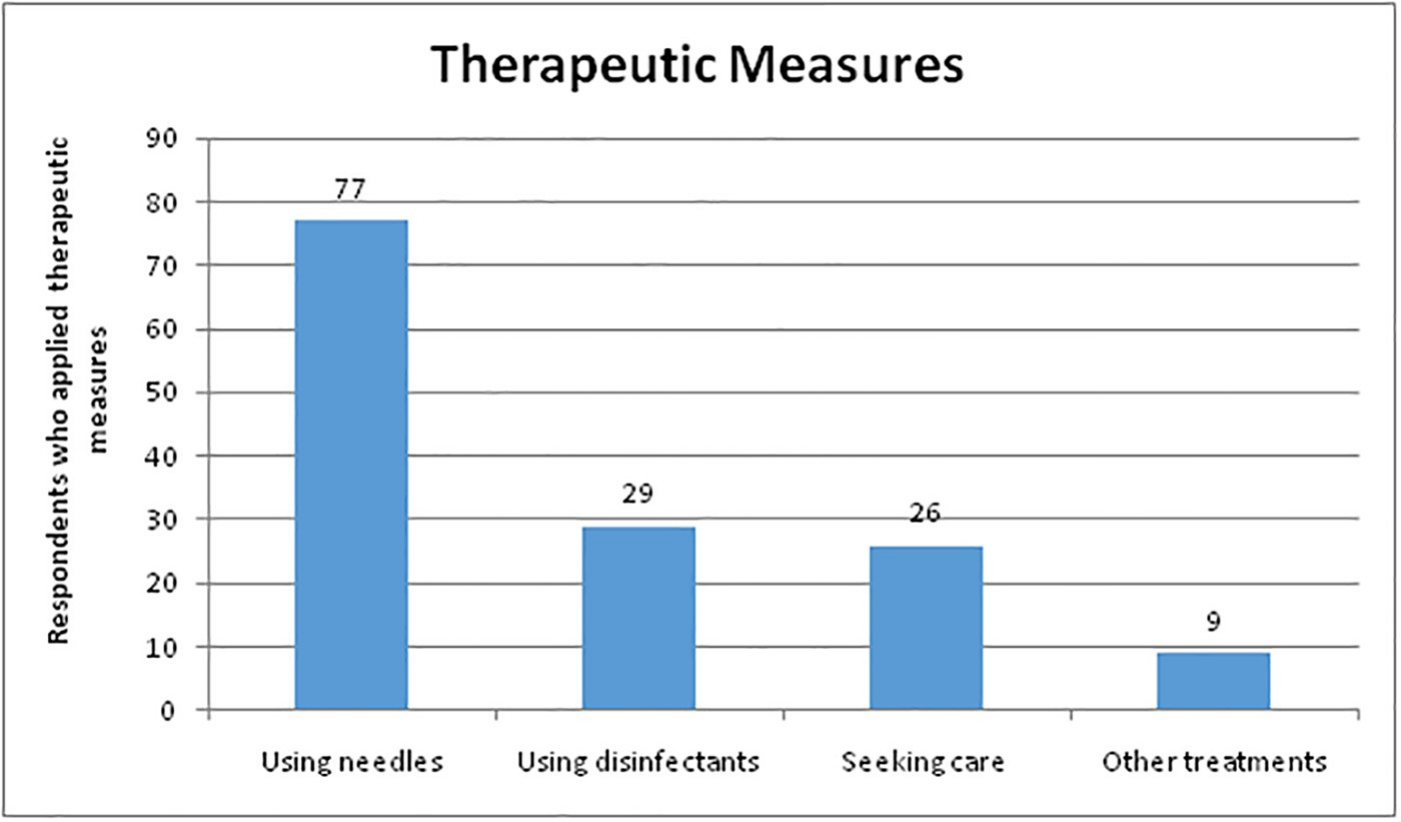
Therapeutic measures taken after a Tungiasis infestation, Limu Saka, Ethiopia, 2021.

Furthermore, 77 (55.7%) of the participants used needles or prickles to extract the *Tunga penetrans* that had been embedded in their skin, while 9% used alternative treatments such as prayer oil, cold hydrotherapy, and Ocimum lamifolium leaves (an Ethiopian traditional medicine used to treat a range of inflammatory diseases) (Fig 1).

### Factors associated with *T*.*penetrans* infestation

Each variable was evaluated using bivariate logistic regression, and variables with a p-value of less than 0.25 were fitted to the multivariable logistic regression. In the multivariable regression, mud-plastered walls, owning cats, having no separated sleeping quarters for domestic animals, travelling >30 minutes to school, and utilizing self-supplied water were all found to be associated with *T*.*penetrans* infestation.

Children who resided in houses with smeared mud walls were 5 times more likely to be infested with *T*.*penetrans* than those who lived in homes with cement-plastered walls [AOR: 5.83, 95% CI(3.44–9.88)]. Children who lived in households with cats were 5.91 times more likely to have *T*.*penetrans* infestation [AOR: 5.91, 95% CI (3.51–10.11)] than children who did not. Infestation with *T*.*penetrans* was also 4.6 times more common in children who resided in homes without a separate living place for their herd animals [AOR: 4.60, 95% CI (2.69–7.86)].

Similarly, children whose parents used self-supplied water were 6.3 times more likely than those whose parents used tap water or public wells to contract the ectoparasite [AOR: 6.30, 95% CI(3.33–11.93)]. *T*.*penetrans* infestation was also associated with the distance between home and school. Children who walked more than 30 minutes to and from school were 2.3 times more likely to get Tungiasis than those who walked less [AOR: 2.37, 95% CI(1.48–3.80)] (Table 5).

**Table 5:** Multivariable logistic regression of factors associated with *T*.*penetrans* infestation among 5-14-year olds, Limu Saka, Ethiopia, 2021.

Variable	*T*.*penetrans* infestation		COR(95% CI)	AOR(95% CI)	P-value
	Yes	No			
**Materials of House wall**					
Smeared mud	132 (30.5%)	301 (69.5%)	3.5 (1.46–8.39)	5.83 (3.44–9.88)	0.001*
Stone/cement	6 (11.1%)	48 (88.9%)	1	1	
**Water source**					
Self-supplied water	39 (58.2%)	28 (41.8%)	4.79 (2.79–8.22)	6.30 (3.33–11.93)	0.001**
Community tap/wells	9 (45.0%)	11 (55.0%)	2.81 (1.13–7.01)	3.61 (1.30–10.02)	0.014*
Piped into dwelling	90 (22.5%)	310 (77.5%)	1	1	
**Presence of animals in thecompound**					
**Cats**					
Yes	59 (53.6%)	51 (46.4%)	4.37 (2.65–7.12)	5.91 (3.51–10.11)	0.0001***
No	79 (21.0%)	298 (79.0%)	1	1	
**Share sleeping quarters with animals**					
Yes	46 (56.1%)	36 (43.9%)	4.34 (2.65–7.12)	4.60 (2.69–7.86)	0.0001***
No	92 (22.7%)	313 (77.3%)	1	1	
**The sleeping situation of child**					
Floor	48 (35.0%)	89 (65.0%)	1.5 (1.01–2.38)	2.00 (0.255–3.66)	0.061^+^
Raised bed	90 (25.7%)	260 (74.3%)	1	1	
**Walk time to school**					
>30 minutes	72 (37.5%)	120 (62.5%)	2.08 (1.39–3.10)	2.37 (1.48–3.80)	0.0001***
<30 minutes	66 (22.4%)	229 (77.9%)	1	1	
**Maternal educational status**					
No formal education	47 (22.7%)	160 (77.3%)	0.75 (0.32–1.59)	0.61 (0.25–1.47)	0.278
Primary schooling (grades 1–8)	40 (30.1%)	93 (69.9%)	1.1 (1.05–2.3)	0.95 (0.38–2.39)	0.926
Secondary schooling (grades 9–10)	39 (37.5%)	65 (62.5%)	1.5 (0.74–3.36)	2.4 (0.98–6.03)	0.053^+^
Vocational training and above*	12 (27.9%)	31 (72.1%)	1		

+Significant in the bivariate analysis *Significant in the multivariable analysis

* = significant association; significant at, * *p< = 0*.*05*, ** p< = 0.*01*, *** p< = 0.*001*

## Discussion

In this study, the overall prevalence of *T*.*penetrans* infestation among primary school students was 28.3%, with a 95% CI (24.2%-32.2%). Other studies conducted in Southern Ethiopia (34.87%) [18], Kenya (48.0%), and Tanzania (97.0%) [19] reported a lower prevalence. This finding, on the other hand, was higher than those found in Kenya (19.1%) [2], Rwanda (25.0%) [11], Nigeria (24.4%) [20], and Cameroon (23%) [3]. Bioclimatic conditions may have a role in the discrepancies in reported prevalence. It’s also important to keep in mind that different studies may employ different epidemiologic case definitions.

Human infections of *T*.*penetrans* have been linked to important socio-cultural features associated with poverty in previous studies [21, 22]. Similarly, in Limu Saka, mud plastered walls, having cats in the household, sharing sleeping quarters with animals, walking >30 minutes to school, and using self-supplied water were all linked to *T*.*penetrans* infestation.

The most frequent style of home in rural Africa is the mud house, which is made up of a simple wattle and daub structure with a thatched roof [23]. In the present study, children who lived in houses with mud-plastered walls were 5.83 times more likely to be infested with *T*.*penetrans* than those who lived in homes with cement walls [AOR: 5.83, 95%CI(3.44–9.88)]. The trickling of sand and dust from mud walls creates ideal conditions for the off-host life cycle of sand fleas in cracks of the floor. Building walls of stone or cement would reduce the prevalence of Tungiasis [24].

Domestic, synanthropic, and sylvatic animals serve as reservoirs for human infestation [6, 25]. Similarly, in the current study, *T*.*penetrans* infestation was 5.9 times more likely in children who lived with cats than in children who did not [AOR: 5.91, 95% CI (3.51–10.11)]. Moreover, children from households without separate sleeping quarters for their domestic animals were 4.6 times more likely to have Tungiasis than those from households with separate sleeping quarters for their domestic animals [AOR: 4.60, 95% CI (2.61–7.86)]. To address this, integrated control strategies in both human and animal reservoirs, in view of a One Health approach, are required.

A further significant determinant of Tungiasis in this study was the length of commute to school. Children who walked>30 minutes each way to and from school were 2 times more likely to develop Tungiasis than those who had a shorter commute [AOR: 2.2, 95% CI (1.48–3.80)]. Long walks to school disorganize children’s attention to ambient stimuli; some of them arrive at their destination sweaty, agitated, and drained, both physically and mentally. This jeopardizes their cleanliness. Weariness is a significant factor that can impair health and hygiene. People less inclined to focus on their cleanliness when they are tired [26].

Furthermore, because it is uncomfortable to walk long distances in closed shoes in hot weather, most children prefer to wear open (less protective) styles of footwear or walk barefoot in many cases [27], thus long walks to school may be a key facilitator of sand flea exposure for many in the context of endemic poverty.

Rural Ethiopia has the poorest access to safe drinking water in Sub-Saharan Africa, with 57% of rural homes using unimproved water [28]. In the current study, children whose parents/guardians used self-supplied water were 6 times more likely to get an infestation than those whose parents used tap water [AOR: 6.03, 95% CI (3.33–11.93)]. Similarly, children living in households that use communal water sources were also 3.6 times more likely to get Tungiasis than those who had piped water delivered to their homes [AOR: 3.61, 95% CI (1.30–10.02)]. Water supply availability and water point functionality are major concerns to maintaining hygienic standards as attention switches to the sustainability of rural water systems. Water, sanitation, and hygiene have all been linked to child health in previous research [29].

### Limitations

Since this is a single-district study, the results are unlikely to be indicative of all other districts. The Physical examination was deemed diagnostic for parasitological characteristics of Tungiasis based on clinical skin lesions observed during the inspection rather than an epiluminescence microscopy (dermatoscope) examination. Although the magnifying lens allows for skin lesions to be examined without being obstructed by skin surface lesions, such equipment was not readily available. It should also be noted that animals were not examined for infection in this study; rather they were only observed as present in the compound and reported as to where they sleep at night.

Finally, while these findings may provide further insights regarding the prevalence and associated factors of Tungiasis among children living in comparable environments, it is necessary to perform future studies with a more structured sampling method that allows for the synthesis of larger sample sizes. Similarly, in order to estimate the burden of and factors related with secondary complications of Tungiasis, additional research is needed in the future.

## Conclusion

Tungiasis was prevalent in 28.3% of the study population (95% CI: 24.2%, 32.2%). Mud plastered homes, sharing sleeping quarters with animals; having a cat in the house; travelling> 30 minutes to school, and using self-supplied water were all linked to Tungiasis. In one way or another, several of the identified factors were linked to poverty. Enhancement of house wall plastering material, fumigation of mud-plastered houses, dusting or spraying insecticides on domestic animals (such as cats), improved access to water, community education about keeping animals separated from living spaces, and hygiene promotion are all needed, with a focus on locally available, low-cost technologies that the poorest families can afford.

## Supporting information

S1 File Questionnaire and observational checklist. (DOCX)

## Abbreviations

AOR: Adjusted odds ratio
COR: Crude odds ratio
Kcal: Kilocalorie
SPSS: Statistical package for social sciences
*T.penetrans*: Tunga penetrans
VIF: Variance inflation factor

## References

[1] Heukelbach J, Jackson A, Ariza L, Calheiros C, Soares V, Feldmeier H. Epidemiology and clinical aspects of tungiasis (sand flea infestation) in Alagoas State, Brazil. J Infect Dev Countries. 2007;1:202–9.

[2] Kamau TM, Ngechu RN, Haile ZT, Mwitari J. An exploration of factors associated with jigger infestation (Tungiasis) among residents of Muranga North District, Kenya. International Journal of Health Sciences and Research. 2014;4(3):1–8.

[3] Collins G, McLeod T, Konfor NI, Lamnyam CB, Ngarka L, Njamnshi NL. Tungiasis: a neglected health problem in rural Cameroon. International Journal of Collaborative Research on Internal Medicine and Public Health. 2009;1(1):2–10.

[4] Ariza L, Wilcke T, Jackson A, Gomide M, Ugbomoiko U, Feldmeier H, et al. A simple method for rapid community assessment of tungiasis. Tropical Medicine & International Health. 2010;15(7):856–64. doi: 10.1111/j.1365-3156.2010.02545.x 20497406

[5] Franck S, Feldmeier H, Heukelbach J. Tungiasis: more than an exotic nuisance. Travel Medicine and Infectious Disease. 2003;1(3):159–66. doi: 10.1016/j.tmaid.2003.09.005 17291909

[6] Muehlen M, Feldmeier H, Wilcke T, Winter B, Heukelbach J. Identifying risk factors for tungiasis and heavy infestation in a resource-poor community in northeast Brazil. Transactions of the Royal Society of Tropical Medicine and Hygiene. 2006;100(4):371–80. doi: 10.1016/j.trstmh.2005.06.033 16297946

[7] Ugbomoiko US, Ariza L, Babamale AO, Heukelbach J. Prevalence and clinical aspects of tungiasis in south-west Nigerian schoolchildren. Tropical doctor. 2017;47(1):34–8. doi: 10.1177/0049475516657503 27402650

[8] Waruguru C, Mwaniki P, Karama M, Muthami L. Prevalence of tungiasis and its associated factors among residents of Kipkelion west sub-county; Kericho county, Kenya. Int J Health Sci Res. 2015;5(8):434–45.

[9] Heukelbach J, Ugbomoiko U. Tungiasis in the past and present: A dire need for intervention. Nigerian Journal of Parasitology. 2007;28(1):1–5.

[10] Central Statistical Agency (CSA) [Ethiopia] and ICF. Ethiopia Demographic and Health Survey 2016. Addis Ababa, Ethiopia, and Rockville, Maryland, USA: CSA and ICF; 2016.

[11] Nsanzimana J, Karanja S, Kayongo M, Nyirimanzi N, Umuhoza H, Murangwa A, et al. Factors associated with tungiasis among primary school children: a cross-sectional study in a rural district in Rwanda. BMC public health. 2019;19(1):1–9. doi: 10.1186/s12889-018-6343-3 31464600 PMC6716852

[12] Assefa Y, Gelaw YA, Hill PS, Taye BW, Van Damme W. Community health extension program of Ethiopia, 2003–2018: successes and challenges toward universal coverage for primary healthcare services. Globalization and Health. 2019;15(1):1–11. 30914055 10.1186/s12992-019-0470-1 PMC6434624

[13] Eisele M, Heukelbach J, Van Marck E, Mehlhorn H, Meckes O, Franck S, et al. Investigations on the biology, epidemiology, pathology and control of Tunga penetrans in Brazil: I. Natural history of tungiasis in man. Parasitology research. 2003;90(2):87–99. 12756541 10.1007/s00436-002-0817-y

[14] Enwemiwe VN, Ojianwuna CC, Anyaele OO. Intensity and clinical morbidities of tungiasis in an impoverished south-west Nigerian community. Parasite epidemiology and control. 2021:e00215. doi: 10.1016/j.parepi.2021.e00215 34124398 PMC8173311

[15] Eyasu AM. Determinants of poverty in rural households: Evidence from North-Western Ethiopia. Cogent Food & Agriculture. 2020;6(1):1823652.

[16] Tamene A, Afework A. Exploring barriers to the adoption and utilization of improved latrine facilities in rural Ethiopia: An Integrated Behavioral Model for Water, Sanitation and Hygiene (IBM-WASH) approach. PloS one. 2021;16(1):e0245289. doi: 10.1371/journal.pone.0245289 33428677 PMC7799797

[17] Tamene A. A Qualitative Analysis of Factors Influencing Household Water Treatment Practices Among Consumers of Self-Supplied Water in Rural Ethiopia. Risk management and healthcare policy. 2021;14:1129. doi: 10.2147/RMHP.S299671 33758565 PMC7981144

[18] Walker SL, Lebas E, De Sario V, Deyasso Z, Doni SN, Marks M, et al. The prevalence and association with health-related quality of life of tungiasis and scabies in schoolchildren in southern Ethiopia. PLoS neglected tropical diseases. 2017;11(8):e0005808. doi: 10.1371/journal.pntd.0005808 28771469 PMC5557602

[19] Mazigo H, Bahemana E, Konje E, Dyegura O, Mnyone L, Kweka E, et al. Jigger flea infestation (tungiasis) in rural western Tanzania: high prevalence and severe morbidity. Transactions of the Royal Society of Tropical Medicine and Hygiene. 2012;106(4):259–63. doi: 10.1016/j.trstmh.2011.12.001 22305586

[20] Ugbomoiko US, Ofoezie IE, Heukelbach J. Tungiasis: high prevalence, parasite load, and morbidity in a rural community in Lagos State, Nigeria. International journal of dermatology. 2007;46(5):475–81. doi: 10.1111/j.1365-4632.2007.03245.x 17472674

[21] Njau NN, Wanzala P, Mutugi M, Ariza L, Heukelbach J. Tungiasis (jigger infestation) in rural Kenya, an emerging infectious disease. Retrovirology. 2012;9(1):1-.22214232

[22] Feldmeier H, Heukelbach J, Ugbomoiko US, Sentongo E, Mbabazi P, von Samson-Himmelstjerna G, et al. Tungiasis—a neglected disease with many challenges for global public health. PLoS neglected tropical diseases. 2014;8(10):e3133. doi: 10.1371/journal.pntd.0003133 25356978 PMC4214674

[23] Adebowale SA, Morakinyo OM, Ana GR. Housing materials as predictors of under-five mortality in Nigeria: evidence from 2013 demographic and health survey. BMC pediatrics. 2017;17(1):1–13. doi: 10.1186/s12887-016-0759-7 28103828 PMC5248529

[24] Elson L, Wiese S, Feldmeier H, Fillinger U. Prevalence, intensity and risk factors of tungiasis in Kilifi County, Kenya II: Results from a school-based observational study. PLoS neglected tropical diseases. 2019;13(5):e0007326. doi: 10.1371/journal.pntd.0007326 31095558 PMC6522002

[25] Mutebi F, von Samson-Himmelstjerna G, Feldmeier H, Waiswa C, Bukeka Muhindo J, Krücken J. Successful treatment of severe Tungiasis in pigs using a topical aerosol containing Chlorfenvinphos, Dichlorphos and gentian violet. PLoS neglected tropical diseases. 2016;10(10):e0005056. doi: 10.1371/journal.pntd.0005056 27727268 PMC5058476

[26] Asawa K, Sen N, Bhat N, Tak M, Sultane P, Mandal A. Influence of sleep disturbance, fatigue, vitality on oral health and academic performance in indian dental students. Clujul medical (1957). 2017;90(3):333–43. doi: 10.15386/cjmed-749 28781530 PMC5536213

[27] Tora A, Tadele G, Aseffa A, McBride CM, Davey G. Health beliefs of school-age rural children in podoconiosis-affected families: A qualitative study in Southern Ethiopia. PLoS neglected tropical diseases. 2017;11(5):e0005564. doi: 10.1371/journal.pntd.0005564 28542227 PMC5444591

[28] Geremew A, Mengistie B, Mellor J, Lantagne DS, Alemayehu E, Sahilu G. Appropriate household water treatment methods in Ethiopia: household use and associated factors based on 2005, 2011, and 2016 EDHS data. Environmental health and preventive medicine. 2018;23(1):1–11. doi: 10.1186/s12199-017-0690-z 30261840 PMC6161466

[29] Ntakirutimana T, O’Connell B, Quinn M, Scheuerman P, Kwizera M, Sunday FX, et al. Linkage between water, sanitation, hygiene, and child health in Bugesera District, Rwanda: a cross-sectional study. Waterlines. 2021;40(1):44–60.

