# Supplementary material for: Prevalence and associated factors of Tunga penetrans infestation among 5-14-year-olds in rural Ethiopia
Source: PLoS One. 2021 Oct 29;16(10):e0259411. doi: 10.1371/journal.pone.0259411 (PMC8555830; doi:10.1371/journal.pone.0259411)
Supplement: S1 File — (DOCX) [file pone.0259411.s001.docx]

# S1 File. Questionnaire and Observational checklist

[Informed Consent Form for _________________________________]

This informed consent form is for parents/guardians in Limu Saka who we are inviting to participate in a research, titled " **A cross-sectional analysis of factors associated with Tunga Penetrans infestation among 5-14-year-olds in rural Ethiopia**``

Aiggan Tamene

Wachemo University

This Informed Consent Form has two parts:

• Information Sheet (to share information about the study with you)

• Certificate of Consent (for signatures if you choose to participate)

You will be given a copy of the full Informed Consent Form

Part I: Information Sheet

My name is Aiggan Tamene and I am an Instructor at Wachemo University, College of Public Health. I would like to invite you to participate in a research topic entitled ‘**A cross-sectional analysis of factors associated with Tunga Penetrans infestation among 5-14-year-olds in rural Ethiopia’**. Your participation is entirely voluntary, and you may choose not to participate. I am going to give you information and invite you to be part of this research. You do not have to decide today whether or not you will participate in the research. Before you decide, you can talk to anyone you feel comfortable with about the research. This consent form may contain words that you do not understand. Please ask me to stop as we go through the information and I will take time to explain. If you have questions later, you can ask them of me or of another researcher

Purpose of the research

The Limu Saka district is one of the largest districts in Western Ethiopia. Anecdotal evidence suggests that Tunga penetrans infestation is still a major nuisance for the local population. Little is known, however, about the prevalence and underlying correlates of the infestation, particularly among children, who are an especially vulnerable group in the region.

Type of Research Intervention

This research will involve your participation in an interview that will take 15-20minutes.

Participant Selection

You are being invited to take part in this research because we feel that your experience as a (mother, a father) can contribute much to our understanding and knowledge of local health practices.

Voluntary Participation

Your participation in this research is entirely voluntary. It is your choice whether to participate or not. You may change your mind later and stop participating even if you agreed earlier

Procedures

During the interview, I or another interviewer will sit down with you in a comfortable place. If it is better for you, the interview can take place in your home. If you do not wish to answer any of the questions during the interview, you may say so and the interviewer will move on to the next question. No one else but the interviewer will be present unless you would like someone else to be there. The information recorded is confidential, and no one else except [name of person(s)] will access to the information documented during your interview.

Risks

There is a risk that you may share some personal or confidential information by chance, or that you may feel uncomfortable talking about some of the topics. However, we do not wish for this to happen. You do not have to answer any question if you feel the question(s) are too personal or if talking about them makes you uncomfortable.

Benefits

There will be no direct benefit to you, but your participation is likely to help us find out more about how to prevent Tungiasis in your community.

Sharing the Results

Nothing that you tell us today will be shared with anybody outside the research team, and nothing will be attributed to you by name. The knowledge that we get from this research will be shared with you and your community before it is made widely available to the public. Each participant will receive a summary of the results. There will also be small meetings in the community and these will be announced. Following the meetings, we will publish the results so that other interested people may learn from the research.

Right to Refuse or Withdraw

You do not have to take part in this research if you do not wish to do so, and choosing to participate will not affect you in any way.

Who to Contact

If you have any questions or concerns about the study, please feel free to contact Aiggan Tamene at 09-13-99-45-30, apublic22@gmail.com. You may also contact Wachemo University Institutional Review Board for answers to questions about subject’s rights. Your willingness to assist with this project is deeply appreciated.

Part II: Certificate of Consent

If the participant is illiterate but gives oral consent, a witness must sign. A researcher or the person going over the informed consent must sign each consent form. I have read the foregoing information, or it has been read to me. I have had the opportunity to ask questions about it and any questions I have been asked have been answered to my satisfaction. I consent voluntarily to be a participant in this study

Print Name of Participant__________________

Signature of Participant ___________________

Date ___________________________

Day/month/year

If illiterate ^[[1]](#footnote-1)^

I have witnessed the accurate reading of the consent form to the potential participant, and the individual has had the opportunity to ask questions. I confirm that the individual has given consent freely.

Print name of witness____________ Thumb print of participant

Signature of witness _____________

Date ________________________

Day/month/year

Statement by the researcher/person taking consent

I have accurately read out the information sheet to the potential participant, and to the best of my ability made sure that the participant understands that the following will be done

1. They will take part in the study

3. Their response will be anonymised and used for the study

I confirm that the participant was given an opportunity to ask questions about the study, and all the questions asked by the participant have been answered correctly and to the best of my ability. I confirm that the individual has not been coerced into giving consent, and the consent has been given freely and voluntarily.

A copy of this ICF has been provided to the participant.

Print Name of Researcher/person taking the consent________________________

Signature of Researcher /person taking the consent__________________________

Date _______________________

Study title:  **A cross-sectional analysis of factors associated with Tunga Penetrans infestation among 5-14-year-olds in rural Ethiopia**

| 1.Sex of the child | Male  Female |
| --- | --- |
| 2.Age of the child (years) | _____________ |
| 3.Respondent | Mother  Father |
| 4.Maternal education | No schooling  Primary  Secondary  University  Other (specify)____________________ |
| 6.Tungiasis status in a child | Infected  Non-infected |
| 7.Clinical manifestations suggesting tungiasis infestation | - Skin lesions (nodules with black centres, suppurative ulcers or punctiform cavities, itching spots, walking difficulty, oedema and skin redness around lesions, loss of toenails or deformed nails, other to be specified):  ___________________________________________________________  ___________________________________________________________  ___________________________________________________________  - Location of skin lesions (feet, toes, fingers,interdigital spaces, other to be specified): ____________________________________________________________  ____________________________________________________________  ________________________________________________________  - History of clinical manifestations (duration in days or weeks or months, chronology of manifestations, other people having the same manifestations at home, similar manifestations in the past, etc.):  ____________________________________________________________  ____________________________________________________________  ____________________________________________________________  ____________________________________________________________  ____________________________________________________________  - Therapeutic measures taken (removal of tunga penetrans using a needle or thorn, use of desinfectant on lesions, seeking care at a health facility, other forms of treatment used to be specified). ____________________________________________________________  ____________________________________________________________  ____________________________________________________________  - Evolution of lesions (improved/healed lesions, sequellae such as nails deformity, etc.):  _________________________________________  _____________________________________________________________  _____________________________________________________________ |
| 8. Body hygiene | Clean feet  Dirty feet (unwashed, covered by dust) |
| 9. Clothes hygiene | Clean clothes  Dirty clothes (unwashed, covered by dust) |
| 10. Wearing shoes | Alwayswear shoes (at home or elsewhere)  Irregular wearing of shoes (only when going to school,to the church, or other social events)  Never wear shoes |
| 11. Possession of domestic animals at home | Yes  No  If yes, specify the type and number of animals (goat, cow, pig, hens, rabbit, cats, dog, etc.):  _____________________________________________ |
| 12. Sharing house with domestic animals | Yes  No  If yes, specify which type of domestic animals:  ____________________________________ |
| 13. Plastering of the house floor | Earthen floor  Yes  No  Cemented floor  Yes  No  Other  Please, specifyother material covering the floor if any:  ____________________________________________________________ |
| Walk time to school |  |
| Family size |  |
| Family Income |  |
| Materials of House roof | Thatched  Corrugated sheet |
| Household waste disposal | Open dumping  Burn/burry  Use a collection service |
| Household latrine facility | Open defecation  Non-improved latrine  Improved, shared latrine  Improved, not shared latrine |
| Water source | Piped into dwelling  Community tap/ wells  Self-supplied |
| Time travelled to fetch water | <30 minutes  >30 minutes |
| Share living space with domestic animals | Yes  No |
| Footwear habit | Never (always barefoot)  Sometimes  Always |
| Feet washing | > 2 times a day  Once per day  Less than once per day |
| Feet washing with soap | Never  Sometimes  Always |
| Shoes worn to school (during the classroom observation) | Closed shoes  Open shoes  No shoes |

**Observational checklist in the primary schools**

| Variables | Category | $(\surd$)Thick when appropriate |
| --- | --- | --- |
| Classroom floor material | Natural soil |  |
|  | Smeared mud |  |
|  | Cracked concrete |  |
|  | Smooth concrete |  |
| Roofing material of classrooms | Corrugated roof |  |
|  | Grass roof/thatched |  |
| Class room wall plastering | Smooth brick/ cement |  |
|  | Cracked brick/ cement |  |
|  | Smeared mud |  |

1. A literate witness must sign (if possible, this person should be selected by the participant and should have no connection to the research team). Participants who are illiterate should include their thumb print as well. [↑](#footnote-ref-1)
